# Musculoskeletal Pain Among School Teachers in Qassim, Saudi Arabia: Prevalence, Pattern, and Its Risk Factors

**DOI:** 10.7759/cureus.17510

**Published:** 2021-08-27

**Authors:** Abdulrhman Aldukhayel, Fatimah K Almeathem, Aram A Aldughayyim, Razan A Almeshal, Emtenan A Almeshal, Jolan S Alsaud, Reema I Albaltan

**Affiliations:** 1 Department of Family and Community Medicine, College of Medicine, Qassim University, Buraydah, SAU

**Keywords:** musculoskeletal pain, teachers, nordic musculoskeletal, örebro musculoskeletal pain screening, back pain, neck pain, qassim, saudi arabia, occupational hazard, school

## Abstract

Introduction

Work-related musculoskeletal disorders (WMSD) are defined as conditions that the environment contributes significantly to / worsens due to work conditions. WMSD comes second in the source of disability in both developed and developing countries. The aim of this study is to measure the prevalence, patterns, and risk factors of musculoskeletal pain disorders among teachers in the Qassim region, Saudi Arabia.

Methods

A cross-sectional study was conducted among school teachers in the Qassim region, Saudi Arabia. A validated online Arabic questionnaire was distributed among teachers living in the Qassim region through multiple social networking applications like Facebook, WhatsApp, and Telegram. The questionnaire consisted of Socio-demographic characteristics, the Nordic Musculoskeletal Questionnaire, and a modified version of the Örebro Musculoskeletal Pain Screening questionnaire.

Results

A total of 503 school teachers were recruited. The proportion of musculoskeletal pain was 91%, and the most common site of pain was back (74.4%), followed by the shoulder (57.5%) and leg (51.5%). The proportion of disabling pain was (80.1%) while the rest was non-disabling pain (19.9%). In the multiple regression model, the predictors of increased disabling pain were back pain, leg pain, neck pain, and those with 51-70 kg weight. In comparison, the predictors of decreased disabling pain were having more than 7 hours of sleep and those with 20-30 classes per week.

Conclusion

Musculoskeletal pain disorders were widely prevalent among school teachers in the Qassim region, Saudi Arabia. Most teachers considered pain as disabling, which negatively affected their attendance. The back, shoulder, and neck were the most painful sites associated with disabling pain.

## Introduction

Work-related musculoskeletal disorders (WMSD) are defined as conditions that the environment contributes significantly to / worsens due to work conditions [1]. WMSD comes second in the source of disability in both developed and developing countries [2]. Musculoskeletal disorders (MSD) include injuries of the muscles, nerves, tendons, cartilages, and spinal disc; examples of these disorders include sprains, back pain, carpal tunnel syndrome, and hernia [1]. However, pain is reported internationally as the most frequent disorder [3]; back pain is more prevalent than others [4]. Many overseas studies showed that almost all workers are experiencing WMSD. A study among therapists in Australia showed that 91% are suffering from pain or discomfort, 48% of these problems concerned their low back and neck [5]. 78.6% of truck drivers and 55.5% of office workers in Qom province, Iran had musculoskeletal disorders [6]. 68.1% of flower farm workers of a study done in Kenya reported musculoskeletal discomfort [2]. In the Kingdom of Saudi Arabia (KSA), the prevalence of lower back pain in different professional groups within a working-age group ranges between 64% and 89% [7]. So as evidence suggests that the development of WMSD is almost inevitable for most workers, it is significantly associated with work organization factors [8].

Furthermore, researchers investigated three main occupational health problems when considering teachers: voice problems, musculoskeletal disorders, and contact dermatitis [9]. They may be at higher risk of suffering from musculoskeletal problems, ranging between 39% and 95% [10] since they might be required to stand for a long time or sit in the wrong posture marking papers or writing for hours [9]. Due to musculoskeletal pain, school teachers suffer a low quality of life, frequent sick leaves, functional impairments, missing out on working days, early retirement, disability, and health cost, eventually affecting the education system [11,12]. In Al-Khobar, eastern Saudi Arabia, a study reported that absenteeism days are positively associated with higher Orebro musculoskeletal pain scores [3]. Many studies showed a negative impact of musculoskeletal pain on the psychological status of the teachers. A survey done among teachers in Penang, Malaysia, found that low back pain is associated with severe depression, extremely intense anxiety [13]. Among Saudi female school teachers, all parts of musculoskeletal pain were statistically substantially correlated with feeling often nervous and lousy mood [11]. Moreover, another Malaysian study demonstrated that psychosocial factors and depression are strong predictors of musculoskeletal pain [12]. 

From a gender perspective, prevalence is higher among female teachers as supported by Sweden and Australian studies; however, a Philipine study denies the significance of gender and back pain. Severity, though, is associated with the female gender [10]. A Chinese study reported that female teachers experienced a higher pain severity in the shoulder than male teachers (93.5 vs. 83.0%; p < .001) [9]. Furthermore, reports also showed a notable link between increasing weight [3,14], the number of children, type of shoes, and the number of teaching years with musculoskeletal pain [3,15]. Regarding the pain sites common in teachers, a Saudi study reported that the lower back came second after shoulder pain and neck pain; lower extremities pain has less prevalence than upper extremities [3]. So many other risks are proved or denied in previous studies; for example, according to a study conducted in Bolivia, rural areas recorded a higher prevalence of musculoskeletal pain than urban areas [16]. In a study carried out in Kenya, some factors showed similar data in both groups with and without low back pain, such as the median age, BMI, working hours, and the number of working years [17]. Moreover, the Aljouf study revealed significant factors associated with increased musculoskeletal pain: age older than 40 years, no exercise, more than ten years of teaching experience, and uncomfortable school furnishings [18].

Many published studies address work-related musculoskeletal disorders; however, these disorders are not fully reported in the region of Qassim. From this point of view, we planned to investigate that problem in a population in this region; and determine the prevalence, pattern, and risk factors associated with it.

## Materials and methods

A cross-sectional study that we carried out among school teachers in Qassim region, Saudi Arabia. To calculate the sample size needed for this study we used a minimum 95% confidence interval and an estimated prevalence of MSDs at 60% based on scientific literature [3,11,18]. Population size, according to the latest census updated by the ministry of education earlier this year, (N): 19,398. Design effect (for cluster surveys-DEFF): 1. The minimum recommended sample size was 362.

We have received a total of 503 convenience responses. A validated online Arabic questionnaire [3] was distributed among teachers living in the Qassim region through multiple social networking applications like Facebook, WhatsApp, and Telegram. The questionnaire consisted of 1) Socio-demographic characteristics: age, weight, marital status, educational level, number of children, sleeping hours, salary, and characteristic of teaching variables including teaching experience years, working daily hours, number of classes, the average number of students per class, and weekly schedule. 2) Nordic Musculoskeletal Questionnaire, a standardized questionnaire for use in epidemiological studies, compares low back, neck, and shoulder pain, used to specify the site of pain. 3) A modified version of the Örebro Musculoskeletal Pain Screening questionnaire to assess the severity of musculoskeletal pain.

The data analyses were performed using Statistical Packages for Social Sciences (SPSS, IBM Corp, Armonk, USA). The physical impact of musculoskeletal pain was assessed using 18 questions adapted from Örebro Musculoskeletal Pain Screening Questionnaire. The questionnaire has been modified into 18 questions with 5-point Likert scale categories ranging from “never” coded as 1 to “always” coded as 5. The score was calculated by adding all 18 questions. A score range from 18 - 90 has been generated, which generally means that the higher the score, the higher the disabling impact experienced by the participants. And by using 50% of the total score point to determine the level of effects, participants were classified with non-disabling pain if the score was below 50% of the total score and above 50% were classified as disabling pain. This criterion has been adapted from the study of Linton et al. [19]. Descriptive statistics had been presented using counts, proportions (%), mean, standard deviation, and median (min-max). Variables were compared to the socio-demographic and musculoskeletal pain characteristics by using the Chi-square test. Significant results presented in the cross-tabulation were then placed in the multiple regression model to determine the significant independent predictors associated with higher physical effects. The odds ratio and 95% confidence interval were also being reported. A p-value cut-off point of 0.05 at a 95% confidence interval was used to determine statistical significance.

We obtained ethical approval from the Regional Research Ethics Committee. The consent of the participants was attached to the inception of the questionnaire.

## Results

A total of 503 school teachers took part in this study. Table 1 presents the socio-demographic characteristics of the teachers. The most common age group was more than 40 years (60.2%), with nearly all (98.2%) working in a governmental school. A significant proportion of them was "married" (86.5%), almost 90% had a university or higher degree. Concerning their weight, more than half (51.5%) were classified into 51-70 kg group, nearly 60% had one to four children, and 31% had more than 20 years in teaching service. Regarding sleeping hours, 77.1% had regular sleeping for 6 to 7 hours, amidst 80.3% having one to four classes per day. Of them, 66.2% were having 10-19 classes per week. In addition, 55.9% worked 3 to 6 hours a day with 20-34 students per class (65.2%). Compared to musculoskeletal pain, the proportion was significantly higher among those working 3 to 6 hours (p=0.001).

**Table 1:** Proportion of musculoskeletal pain according to teachers' socio-demographic characteristics ^§^P-value has been calculated using Chi-square test. **Significant at p<0.05 level.

Study data	Overall, N (%) ^(n=503)^	Having musculoskeletal pain		P-value^§^
		Yes, N (%) ^(n=458)^	No, N (%) ^(n=45)^	
Type of school				
Governmental	494 (98.2%)	451 (98.5%)	43 (95.6%)	0.159
Private	09 (01.8%)	07 (01.5%)	02 (04.4%)	
Age group				
≤40 years	200 (39.8%)	180 (39.3%)	20 (44.4%)	0.501
>40 years	303 (60.2%)	278 (60.7%)	25 (55.6%)	
Marital status				
Unmarried	68 (13.5%)	62 (13.5%)	06 (13.3%)	0.970
Married	435 (86.5%)	396 (86.5%)	39 (86.7%)	
Educational level				
Secondary or diploma	53 (10.5%)	49 (10.7%)	04 (08.9%)	0.706
University or above	450 (89.5%)	409 (89.3%)	41 (91.1%)	
Weight group				
<50 kg	17 (03.4%)	15 (03.3%)	02 (04.4%)	0.601
51 – 70 kg	259 (51.5%)	239 (52.2%)	20 (44.4%)	
71 – 90 kg	160 (31.8%)	142 (31.0%)	18 (40.0%)	
>90 kg	67 (13.3%)	62 (13.5%)	05 (11.1%)	
Number of children				
None	40 (08.0%)	37 (08.1%)	03 (06.7%)	0.543
1 – 4 children	285 (56.7%)	256 (55.9%)	29 (64.4%)	
≥5 children	178 (35.4%)	165 (36.0%)	13 (28.9%)	
Teaching years				
1 – 10 years	114 (22.7%)	101 (22.1%)	13 (28.9%)	0.197
11 – 15 years	123 (24.5%)	115 (25.1%)	08 (17.8%)	
16 – 20 years	110 (21.9%)	104 (22.7%)	06 (13.3%)	
>20 years	156 (31.0%)	138 (30.1%)	18 (40.0%)	
Sleeping hours				
4 – 5	73 (14.5%)	68 (14.8%)	05 (11.1%)	0.391
6 – 7	388 (77.1%)	354 (77.3%)	34 (75.6%)	
>7	42 (08.3%)	36 (07.9%)	06 (13.3%)	
Classes per day				
1 – 4	404 (80.3%)	364 (79.5%)	40 (88.9%)	0.130
5 – 7	99 (19.7%)	94 (20.5%)	05 (11.1%)	
Classes per week				
1 – 9	47 (09.3%)	41 (09.0%)	06 (13.3%)	0.537
10 – 19	333 (66.2%)	303 (66.2%)	30 (66.7%)	
20 – 30	123 (24.5%)	114 (24.9%)	09 (20.0%)	
Daily hours				
3 – 6	281 (55.9%)	245 (53.5%)	36 (80.0%)	0.001 **
7 – 9	222 (44.1%)	213 (46.5%)	09 (20.0%)	
Student per class				
5 – 19	79 (15.7%)	68 (14.8%)	11 (24.4%)	0.132
20 – 34	328 (65.2%)	299 (65.3%)	29 (64.4%)	
≥35	96 (19.1%)	91 (19.9%)	05 (11.1%)	

**Figure 1:**
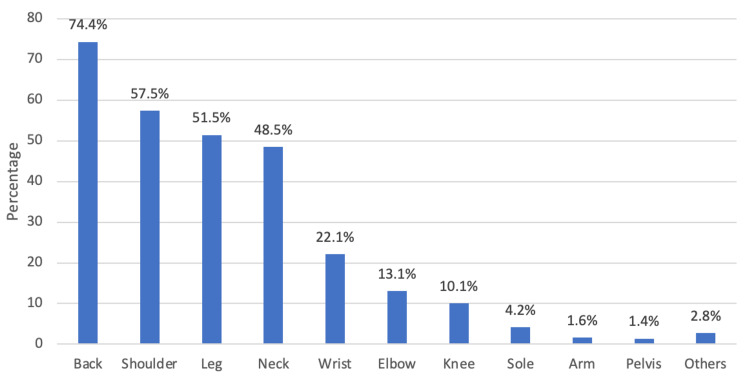
Distribution of site of pain

The characteristics of teachers with musculoskeletal pain are presented in Table 2. It can be observed that the most common site of musculoskeletal pain was the back (74.4%), followed by the shoulder (57.5%) and leg (51.5%), while the least of them was the pelvis (1.4%) (Figure 1). Furthermore, 27.6% of the participants had 1-3 days of absences due to the pain, nearly 80% had pain for more than six months. Additionally, 81.7% indicated that they usually wear medical shoes or without a heel.

**Table 2:** Characteristics of teachers’ musculoskeletal pain (n=503) * Variable with multiple responses.

Variables	N (%)
Site of pain *	
No pain	45 (08.9%)
Back	374 (74.4%)
Shoulder	289 (57.5%)
Leg	259 (51.5%)
Neck	244 (48.5%)
Wrist	111 (22.1%)
Elbow	66 (13.1%)
Knee	51 (10.1%)
Sole	21 (04.2%)
Arm	08 (01.6%)
Pelvis	07 (01.4%)
Others	14 (02.8%)
Days of absenteeism	
None	294 (58.4%)
1 – 3 days	139 (27.6%)
4 – 10 days	54 (10.7%)
>10 days	16 (03.2%)
Duration of pain	
No pain	45 (08.9%)
<3 months	29 (05.8%)
3 – 6 months	29 (05.8%)
>6 months	400 (79.5%)
Type of shoes	
Medical shoes or without heel	411 (81.7%)
With heel and not medical	92 (18.3%)

In determining the long-term disability with musculoskeletal pain, according to the 18 modified Örebro variables, the mean score was 52.9 (SD 11.5). Among 503 research participant teachers, 80.1% were classified into disabling pain and 19.9% with non-disabling pain. Variables indicated a higher frequency of "always" disabling pain: 22.3% of teachers think physical activity always makes the pain worse, and that 19.7% of teachers were satisfied with their work, routines, and salary (Table 3). 

**Table 3:** Assessment of the modified ÖREBRO Musculoskeletal Pain Screening Questionnaire (n=503)

Variables	Never N (%)	Sometimes N (%)	Often N (%)	Usually N (%)	Always N (%)
If you take into consideration your work routines, management, salary, promotion possibilities, and workmates, how satisfied are you with your job?	73 (14.5%)	53 (10.5%)	158 (31.4%)	120 (23.9%)	99 (19.7%)
Physical activity makes my pain worse?	77 (15.3%)	45 (08.9%)	149 (29.6%)	120 (23.9%)	112 (22.3%)

The proportion of disabling pain was more common among those who had a university of higher degree (p=0.047) and those who had sleeping hours of 6 to 7 (p=0.008). In contrast, the proportion was less among those with less than 50 kg weight group (p=0.013), those with 1-9 classes per week (p=0.006), and those with 3-6 daily working hours (p=0.006) (Table 4).

**Table 4:** Physiological Impact of Musculoskeletal pain in relation to socio-demographic characteristics ^§ ^P-value has been calculated using Chi-square test. ** Significant at p<0.05 level.

Factor			P-value ^§^
	Non-disabling pain N (%) ^(n=100)^	Disabling pain N (%) ^(n=403)^	
Educational level			
Secondary or diploma	16 (16.0%)	37 (09.2%)	0.047 **
University or above	84 (84.0%)	366 (90.8%)	
Weight group			
<50 kg	08 (08.0%)	09 (02.2%)	0.013 **
51 – 70 kg	44 (44.0%)	215 (53.3%)	
71 – 90 kg	37 (37.0%)	123 (30.5%)	
>90 kg	11 (11.0%)	56 (13.9%)	
Sleeping hours			
4 – 5	14 (14.0%)	59 (14.6%)	0.008 **
6 – 7	70 (70.0%)	318 (78.9%)	
>7	16 (16.0%)	26 (06.5%)	
Classes per week			
1 – 9	17 (17.0%)	30 (07.4%)	0.006 **
10 – 19	56 (56.0%)	277 (68.7%)	
20 – 30	27 (27.0%)	96 (23.8%)	
Daily hours			
3 – 6	68 (68.0%)	213 (52.9%)	0.006 **
7 – 9	32 (32.0%)	190 (47.1%)	

We also found that the proportion of disabling pain was more common in the back (p<0.001), shoulder (p<0.001), leg (p<0.001), neck (p<0.001), other region pain (p=0.002), and those with more than six months of pain duration (p<0.001). In contrast, the proportion was less among those with more than ten days of absence (p<0.001) (Table 5).

**Table 5:** Physiological Impact in relation to the Characteristics of teachers with musculoskeletal pain * Variable with multiple responses. ^§^ P-value has been calculated using Chi-square test. ** Significant at p<0.05 level.

Factors			P-value ^§^
	Non-disabling pain N (%) ^(n=100)^	Disabling pain N (%) ^(n=403)^	
Site of pain *			
Back	49 (49.0%)	325 (80.6%)	<0.001 **
Shoulder	40 (40.0%)	249 (61.8%)	<0.001 **
Leg	29 (29.0%)	230 (57.1%)	<0.001 **
Neck	27 (27.0%)	217 (53.8%)	<0.001 **
Wrist	15 (15.0%)	96 (23.8%)	0.057
Elbow	10 (10.0%)	56 (13.9%)	0.302
Others	09 (09.0%)	92 (22.8%)	0.002 **
Days of absenteeism			
None	77 (77.0%)	217 (53.8%)	<0.001 **
1 – 3 days	11 (11.0%)	128 (31.8%)	
4 – 10 days	06 (06.0%)	48 (11.9%)	
>10 days	06 (06.0%)	10 (02.5%)	
Duration of pain			
No pain	39 (39.0%)	06 (01.5%)	<0.001 **
<3 months	10 (10.0%)	22 (05.5%)	
3 – 6 months	07 (07.0%)	22 (05.5%)	
>6 months	44 (44.0%)	356 (88.3%)	

In the multiple regression model, we observed that compared to teachers with less than 50 weight, those teachers in the 51-70 weight group were four times higher to be more associated with disabling pain (adjusted odds ratio [AOR]=4.180; 95% CI=1.160-15.062; p=0.029). Furthermore, we observed that teachers who complained of pain in the back (AOR=2.888; 95% CI=1.683-4.956; p<0.001), pain in the leg (AOR=2.167; 95% CI=1.257-3.735; p=0.005), and pain in the neck (AOR=2.224; 95% CI=1.220-4.055; p=0.009) were twice as higher to have disabling pain. On the other hand, teachers who were having slept for more than 7 hours (AOR=0.359; 95% CI=0.160-0.806; p=0.013) and those with 20-30 classes per week (AOR=0.421; 95% CI=0.229-0.772; p=0.005) were significantly less likely to have disabling pain (Table 6).

**Table 6:** Multiple regression analysis to determine the independent significant predictor associated with high physiological impact due to musculoskeletal pain(n=503) AOR – Adjusted Odds Ratio; CI – Confidence Interval. ** Significant at p<0.05 level.

Factor	AOR	95% CI	P-value
Weight group			
<50 kg	Ref		
51 – 70 kg	4.180	1.160 – 15.062	0.029 **
71 – 90 kg	1.237	0.544 – 2.811	0.612
>90 kg	1.300	0.561 – 3.011	0.541
Sleeping hours			
4 – 5	Ref		
6 – 7	0.384	0.141 – 1.043	0.060
>7	0.359	0.160 – 0.806	0.013 **
Classes per week			
1 – 9	Ref		
10 – 19	1.568	0.680 – 3.616	0.292
20 – 30	0.421	0.229 – 0.772	0.005 **
Pain in the back			
No	Ref		
Yes	2.888	1.683 – 4.956	<0.001 **
Pain in the leg			
No	Ref		
Yes	2.167	1.257 – 3.735	0.005 **
Pain in the neck			
No	Ref		
Yes	2.224	1.220 – 4.055	0.009 **

## Discussion

This study was carried out to determine the prevalence of musculoskeletal pain disorders (MSDs) among school teachers, the risk factors, and disabling impact. Among 503 school teachers, the proportion of musculoskeletal pain disorders was 91%, which is higher than the other studies conducted in Saudi Arabia, with a prevalence of musculoskeletal pain varying from 46.1% to 79.2% [3,11,18]. Similarly, in Africa, the 12-month prevalence of MSDs was lower than our report with 57.3% and 64.9%, respectively [12,17]. The reported prevalence of MSDs in Malaysia and India was also lower than our results [13,14]. Incidentally, school teachers in Bolivia had the highest prevalence rate of MSDs with 86% [16], which was in line with our results.

Additionally, teachers most frequently complained site of pain was the back (74.7%), followed by the shoulder (57.5%), leg (51.5%), and neck (48.5%). These findings are consistent with the report of Darwish et al., which indicated that among the 240 female school teachers, lower back pain (63.8%) was the most commonly reported site of pain, followed by the shoulder (45.4%), neck (42.1%), and leg (40%) [3]. Abdel-Salam and colleagues [18] reported pain sites: low back pain (68.4%), knee (58.6%), and neck pains were the most commonly reported MSDs among secondary school female teachers. On the other hand, in Brazil [15], many of the teachers were suffering from either lower limbs (41.1%) or upper limbs (23.7%), while in Bolivia [16], the most common site of pain was the neck (47%) which was the fourth most common site of pain in our study.

Several influential factors have been identified affecting musculoskeletal pain disorders: socio-demographic, environmental, and occupational. Darwish and colleagues noted that increasing age, years of teaching, and weight were associated with more pain [3]. Similarly, Temesgen and associates [12], also documented that teaching experience and regular physical exercise were the factors that significantly influenced shoulder and neck pains. However, in our study, the proportion of MSDs in age, weight, and teaching years were similar across the groups and did not demonstrate as influential factors of musculoskeletal pain. Interestingly, we noted that daily work hours were the only significant factor associated with musculoskeletal pain (p=0.001), also mentioned in a study conducted by Darwish et al. [3]. 

Moreover, based on Örebro's musculoskeletal pain score, the proportion of disabling pain was 80.1%, and the rest were non-disabling pain (19.9%); this result is relatively higher than that of Darwish et al. [3]. We found disabling pain among 53.3% of school teachers, while in a study Abdel-Salam and colleagues [18], the proportion of disabling pain was relatively lower with only 35.6%. On the other hand, Elias et al. [17] indicated that the majority of the primary school teachers in Kenya, detected to have a minimal disability and few of them had a moderate or severe disability due to low back pain.

Furthermore, in our multivariate regression estimates, weight was one of the significant predictors of disabling pain, where the increase of disabling pain was associated with weight 51-70 kg. This finding is consistent with the paper of Darwish et al. [3], as they identified weight as one of the independent factors associated with higher Örebro musculoskeletal pain scores. They further noted that working daily hours and shoe type were other factors that significantly influence pain scores. In the Aljouf region, Saudi Arabia [18], the study suggests that the factors that greatly affected disabling pain were; age more than 40 years, not practicing exercise, more than 10 years of teaching, and uncomfortableness of school furniture. In our study, age group and teaching years were not identified as the influential factors of disabling pain. Conversely, we noted that pain in the back, leg, and neck was the significant independent predictor of disabling pain. On the other hand, having enough hours of sleep and having more classes per week were the independent factors that decreased the risk of disabling pain which could be the protective factors for long-term disability due to pain. Likewise, more than a quarter of teachers reported having 1 to 3 days of absence due to pain. Nearly 80% of teachers complained about pain for more than six months, and these variables showed a significant relationship with disabling pain (p<0.001). Further, literature suggested that disabling pain was significantly associated with increasing teaching years [18,15,20]. However, the findings of this study pointed that years in teaching did not show significant effect with disabling pain which was not consistent with previous reports. The rest of the socio-demographic characteristics did not show considerable risk with disabling pain, including; marital status, the number of children, classes per day, student per class, and the type of shoes (p>0.05).

Limitations

We collected our data through an online survey as the country is following strict regulations due to the COVID-19 pandemic. Respondent biases to these surveys are possible. So generalization of this study's results should be made carefully.

## Conclusions

Musculoskeletal pain disorders were widely prevalent among school teachers in Qassim region, Saudi Arabia. Most teachers considered pain as disabling, which negatively affected their attendance. Back, shoulder, and neck pains were mainly associated with the risk of developing disabling pain. Health awareness programs are essential among school teachers. These programs should include regular exercise, which could be the best method to alleviate pain and suffering, leading to decreased MSDs. More importantly, appropriate measures are necessary to reduce the risk of long-lasting disability. These measures can be associated with the changes in personal and environmental aspects, which will eventually improve their health status.
